# A Genome-Wide Pharmacogenetic Study of Growth Hormone Responsiveness

**DOI:** 10.1210/clinem/dgaa443

**Published:** 2020-07-11

**Authors:** Andrew Dauber, Yan Meng, Laura Audi, Sailaja Vedantam, Benjamin Weaver, Antonio Carrascosa, Kerstin Albertsson-Wikland, Michael B Ranke, Alexander A L Jorge, Jose Cara, Michael P Wajnrajch, Anders Lindberg, Cecilia Camacho-Hübner, Joel N Hirschhorn

**Affiliations:** 1 Division of Endocrinology, Children’s National Hospital, Washington, DC; 2 Division of Endocrinology, Boston Children’s Hospital, and Program in Medical and Population Genetics, Broad Institute, Harvard Medical School, Boston, Massachusetts; 3 Department of Pediatrics, Institut de Recerca (VHIR), Hospital Vall d’Hebron, Centre for Biomedical Research on Rare Diseases (CIBERER), Autonomous University, Barcelona, Spain; 4 Department of Physiology/Endocrinology, Institute of Neuroscience and Physiology, Sahlgrenska Academy, University of Gothenburg, Gothenburg, Sweden; 5 University Children´s Hospital, Paediatric Endocrinology, Tübingen, Germany; 6 Unidade de Endocrinologia do Desenvolvimento (LIM42), Hospital das Clinicas da Faculdade de Medicina da Universidade de Sao Paulo, Sao Paulo, Brazil; 7 Pfizer Inc, Rare Disease, New York; 8 Pfizer, Data Management, Sollentuna, Sweden

**Keywords:** growth hormone, pharmacogenetics, short stature, genome-wide association

## Abstract

**Context:**

Individual patients vary in their response to growth hormone (GH). No large-scale genome-wide studies have looked for genetic predictors of GH responsiveness.

**Objective:**

To identify genetic variants associated with GH responsiveness.

**Design:**

Genome-wide association study (GWAS).

**Setting:**

Cohorts from multiple academic centers and a clinical trial.

**Patients:**

A total of 614 individuals from 5 short stature cohorts receiving GH: 297 with idiopathic short stature, 276 with isolated GH deficiency, and 65 born small for gestational age.

**Intervention:**

Association of more than 2 million variants was tested.

**Main Outcome Measures:**

Primary analysis: individual single nucleotide polymorphism (SNP) association with first-year change in height standard deviation scores. Secondary analyses: SNP associations in clinical subgroups adjusted for clinical variables; association of polygenic score calculated from 697 genome-wide significant height SNPs with GH responsiveness.

**Results:**

No common variant associations reached genome-wide significance in the primary analysis. The strongest suggestive signals were found near the *B4GALT4* and *TBCE* genes. After meta-analysis including replication data, signals at several loci reached or retained genome-wide significance in secondary analyses, including variants near *ST3GAL6*. There was no significant association with variants previously reported to be associated with GH response nor with a polygenic predicted height score.

**Conclusions:**

We performed the largest GWAS of GH responsiveness to date. We identified 2 loci with a suggestive effect on GH responsiveness in our primary analysis and several genome-wide significant associations in secondary analyses that require further replication. Our results are consistent with a polygenic component to GH responsiveness, likely distinct from the genetic regulators of adult height.

Growth disorders resulting in short stature have numerous etiologies, including many different inherited clinical syndromes. Specific genetic etiologies include disorders of the growth hormone–insulin-like growth factor-1 (GH/IGF-1) axis—GH deficiency (GHD) and hormone resistance—and a wide variety of additional genetic syndromes such as Turner syndrome, Noonan syndrome, and skeletal dysplasias (1-3). Other children with short stature are born small for gestational age (SGA) and do not have “catch-up” growth. Finally, the majority of children with short stature have no known specific cause and are classified as idiopathic short stature (ISS).

Recombinant human GH has been used in pediatric populations to increase height for individuals with many of these conditions. There is substantial variability in growth response to GH, not only across types of growth disorders but also between individuals who share the same general etiology for their short stature (4-6). The variable response seen between different disorders suggests that the sensitivity to treatment is associated with the underlying cause of each condition. However, the additional variability within each disorder indicates that individual factors, including possibly genetic variation, also contribute to GH responsiveness.

Previously, disease-specific models have been developed to predict a patient’s response to GH. These models include multiple clinical variables such as parental heights, age at GH initiation, degree of short stature, peak GH levels on stimulation testing, GH dose, and birth parameters (7). However, these clinical parameters only partially predict variation in treatment response. Given the inclusion of parental height (a proxy for genetic determinants of height) in prediction models, and the high heritability of height, it is quite plausible that genetic variation could influence response to GH. Furthermore, identifying genetic variants that influence response could shed light on the biological mechanisms underlying the variability in response to GH therapy.

Several studies have proposed candidate variants in single genes within the GH/IGF-1 axis for influencing GH response. The most widely studied of these is a polymorphism in *GHR* that deletes exon 3 (*GHR*-d3), which has been associated with response to GH therapy in children with short stature. However, this association has only been seen in small samples and has not been consistently replicated (8-12). Similarly, a polymorphism in the IGFBP-3 promoter has been associated with GH response, but this has not been robustly replicated (13, 14). The PREDICT study was designed to take a more comprehensive approach to examining genetic predictors of GH response (15). In that study, a more extensive candidate gene study was performed: 170 patients (110 with GHD and 60 with Turner syndrome) underwent genotyping of approximately 1500 single nucleotide polymorphisms (SNPs) in a list of 103 candidate genes related to growth. Additionally, baseline gene expression analysis was performed in a subset of patients. The genotypes and gene expression profiles were then correlated with first-year GH response. The study identified a total of 11 genes associated with GH responsiveness in patients with GHD and 10 genes in patients with Turner syndrome. However, a subsequent replication study was performed by the same research group in which none of the original SNP signals were reliably replicated (16). There was some suggestive evidence for signals in the *SOS1* and *INPPL1* genes. Ultimately, they concluded that genotype information did not add much to the predictive capability of clinical parameters in determining GH responsiveness. The PREDICT study did identify gene expression profiles associated with both growth response (15) as well as with increase in IGF-1 levels at 1 month of treatment (17). Using bioinformatics techniques, they were able to identify pathways, such as glucocorticoid response, which are potentially involved in GH responsiveness.

To date, there have be no large genome-wide association studies (GWASs) of GH responsiveness, which would provide a comprehensive assessment of the role of common genetic variation in GH response. Recent GWASs of adult height have identified hundreds of associated common variants (18) and, as suggested by the correlation between parental height and GH response, these may also be candidates for influencing GH response and as such merit additional attention. Finally, rarer genetic variants influence height, including those responsible for single-gene disorders (19, 20), and these may also influence GH response. For example, some genetic syndromes (such as short stature homeobox-containing gene deficiency (19) are characterized by better responses to GH than others (such as spondyloepiphyseal dysplasia (20), consistent with the idea that genetic factors may influence GH response.

To search for genetic variation that affects response to GH, we carried out what is to our knowledge the largest GWAS of response to GH, consisting of a total of 614 children including 276 with idiopathic GHD, 297 children with ISS, and 41 children born SGA (with neither GHD nor ISS). We identify several suggestive candidate polymorphisms for influencing GH response and also examine the role of height-associated variants on GH response. Finally, we observe no evidence to support the previously reported association between the *GHR*-d3 or IGFBP-3 promoter polymorphisms and GH response.

## Participants and Methods

### Description of study samples

The study included 614 children who were treated with GH on the basis of the diagnosis of GHD, SGA, or ISS. Individuals were recruited from 5 cohorts including the Boston Children’s Hospital short stature cohort, Barcelona, Sweden, a Pfizer USA clinical trial for patients with ISS, and individuals who were previously collected by KIGS investigators (Pfizer International Growth Database). The majority (504) of these samples are of self-described European ancestry. A replication sample from Brazil consisted of 113 individuals treated with GH for GHD, SGA, or ISS.

The study was approved by the Institutional Review Board of Boston Children’s Hospital as well as by local review boards in Barcelona, Sweden, Pfizer USA, and KIGS (Brazil). Written informed consent was obtained from all patients’ parents or legal guardians.

All clinical information was abstracted from the available medical records/research databases at the respective centers. For all patients, we collected information on birth weight, gestational age, peak GH value during pretreatment testing for GHD, age and height at GH start, average GH dose, parents’ heights, and height at the end of 1 year of GH therapy. Height standard deviation scores (SDSs) were calculated by adjusting for age and gender according to Prader et al (21). Birth weight SDSs were calculated according to Niklasson et al (22). The midparental height (MPH) SDS was calculated by the following: (father’s height SD score + mother’s height SD score) ÷ 1.61 (23). Height gain (delta height) was defined by the difference between height SDS at the end of 1 year of treatment and the height SDS at start of GH therapy.

Patients with known chromosomal disorders or other significant comorbid conditions were excluded from the study. We stratified samples by diagnosis. Patients with a peak GH value below 10 ng/mL were classified as GHD. Due to the nature of the cohort, GH levels were not assayed centrally but rather were based on each center’s reported values. Patients were excluded if they had additional pituitary hormone deficiencies. Patients who had a birth weight SDS below –2 were classified as SGA. Patients could be assigned to both the SGA and GHD categories. Patients who did not meet either of these criteria were classified as ISS. All female patients were required to remain prepubertal throughout the study period, which was defined as Tanner stage 1 breast development. Male participants were required to have testicular volumes ≤6 cc or be documented as Tanner stage ≤2 at study completion. The clinical characteristics of the patients are summarized in Table 1.

**Table 1.** Clinical Characteristics of Participants

	GWAS Cohorts	Replication Cohort
**Individuals**	614	113
**Male**	437	73
**Female**	177	40
**Gestational age (weeks)**	38.6 (2.9)	37.2 (3.1)
**Birth weight (g)**	2969 (693)	2754 (809)
**Birth weight SDS**	–0.70 (1.14)	–0.60 (1.48)
**Midparental height (cm)**	164.6 (5.8)	161.9 (5.35)
**Age at start (years)**	8.14 (2.71)	8.31 (2.55)
**Average growth hormone dose (mg/kg/week)**	0.28 (0.12)	0.29 (0.06)
**Height at start (Prader SDS)**	–3.00 (0.72)	–3.77 (1.18)
**Δ height SDS during first year of therapy**	0.78 (0.40)	0.76 (0.72)
**ISS**	297	42
**GHD**	276	44
**SGA**	65	30
**SGA & GHD**	24	3

The numbers in parens are SD, standard deviations. The “SDS” listed for Birth Weight SDS, Prader SDS and Height SDS are standard deviation scores.

Abbreviations: GHD: growth hormone deficiency; GWAS, genome-wide association study; ISS, idiopathic short stature; SDS: standard deviations above or below the mean; SGA: small for gestational age; SGA_SDS, standard deviation score for being small for gestational age.

### Genotyping and quality control

Samples were genotyped using either a combination of the Illumina HumanOmni1-Quad BeadChip (HO) and HumanExome (HE) BeadChip platforms, or on the HumanOmniExpressExome (HOEE) BeadChip v1.0 or v1.2 platforms; all of these platforms cover a similar genome-wide set of common variants and a more comprehensive set of exonic variants. We used Illumina’s GenCall algorithm to generate genotype calls for all platforms. For the rarer exonic variants, we generated a second set of genotype calls using zCall (24), which is specifically designed for calling rare SNPs from array-based platforms.

Quality control was performed separately for each of the platforms (HO, HE, and HOEE). The minimum overall call rate for passing samples was 95%. Samples were also removed for being outliers for amount of heterozygosity, mismatch between reported gender and genotypic data, or close relationship (estimated genomic identity by descent ≥0.2) with other samples. Self-reported ancestry and gender were compared with ancestry and sex directly estimated from the genetic data; discordant samples were removed. Standard additional filters were applied to remove poorly genotyped SNPs, including variant call rate <95%, Hardy-Weinberg equilibrium *P* value < 10^–6^, and evidence of nonrandom missingness or batch effects. In total, 588 individuals had genotype data passing quality control and the required phenotypic information for association analysis.

### Genotype imputation

To expand the coverage of the genotyping platforms to include the vast majority of common variants, we imputed genotypes for all polymorphic markers in the 1000 Genomes Phase I Integrated Release Version 3 (March 2012) (25), using the program IMPUTE2 (26, 27), thereby generating datasets of 36 388 217 autosomal and 1 250 157 sex chromosomal variants. The data from HO and HE platforms were merged and then imputed; the remaining samples genotyped on the HOEE platform were imputed separately. We removed SNPs with a low quality of imputation (info score <0.4).

### Association analysis

**Figure 1.**
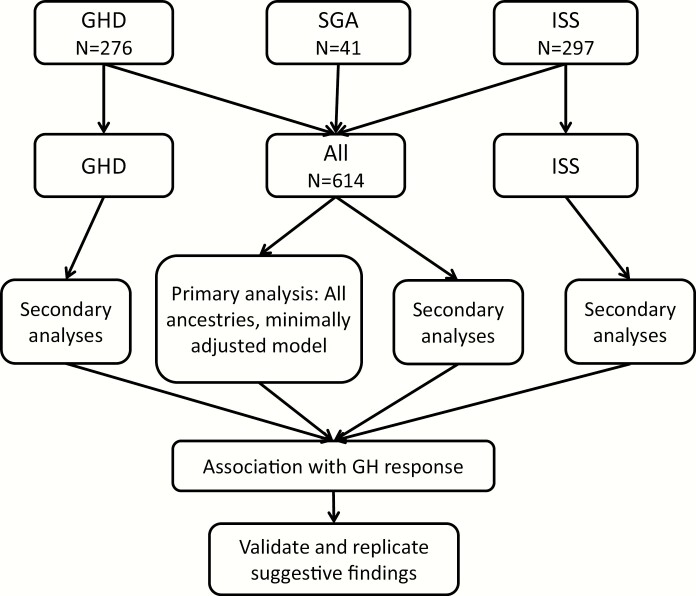
Analysis plan for testing association to growth hormone response. The primary analysis was to combine patients in all 3 categories: growth hormone deficiency (GHD), small for gestational age (SGA) and idiopathic short stature (ISS), including all ancestries and performing a minimally adjusted analysis (age, sex, and principal components of genetic ancestry). Secondary analyses included testing GHD or ISS alone, limiting the analyses to individuals of European ancestry and adjusting for additional covariates. Additional covariates were gestational age in weeks, birthweight standard deviation score (SDS), age at GH start, average GH dose, height SDS (Prader) before treatment, average height of parents, and severity of GHD. For principal components of ancestry, the top 10 eigenvalues were used for all ancestry analyses and the top 3 eigenvalues for analyses of European ancestry samples. For single-variant analysis, associations were considered to have genome-wide significance if they reached *P < *5 × 10^–8^ in the primary analysis; suggestive associations were those that reached *P < *5 × 10^–7^ in the primary or secondary analyses.

The primary outcome was the change in height SDS; we performed an inverse normal transformation to ensure normality of the outcome variable, but results were similar if this transformation was omitted. We carried out association testing between this outcome and SNP genotype (coded as allele dosage between 0 and 2), using an additive model in a regression framework implemented in PLINK (28). We performed analyses stratified by diagnosis (all diagnoses, ISS only, and GHD only; the number of SGA samples was too small for reliable analyses) and by ancestry (all ancestries and European ancestry only). For each analysis, we used 2 different models: a minimally adjusted model (age and gender included as covariates) and a maximally adjusted model (age; gender; gestational age; birth weight SDS; age at start of GH therapy; GH dosage; height SDS at start of GH therapy; MPH; and whether the patient has mild or severe GHD, defined as a peak GH level <5, included as covariates). In addition, principal components (29) were included as covariates to account for ancestry; for the multiethnic analysis, 10 principal components were included; for European samples, 3 principal components were included. The primary analysis was considered to be all ancestries, all diagnoses, and a minimally adjusted model (Fig. 1).

To account for the potential confounding effect due to different genotyping platforms, the analyses were performed separately for the Pfizer USA samples genotyped on HO/HE and all other samples genotyped on HOEE, and results were meta-analyzed. For meta-analysis, we used the inverse-variance fixed effects method to combine the results, using METAL (30), weighed by standard error. Genome-wide significance for our primary analysis was set at *P < *5 × 10^-8^.

### Association analysis of low-frequency coding variants

For rarer (minor allele frequency <5%) coding variants represented on the exome chip, 2 sets of analyses were performed using rare variant tests (31). We performed association analysis of each variant individually (single-variant analysis) and aggregate analysis of missense, splice, and loss-of-function variants within each gene (gene-based analysis). Gene-based analyses were performed with sequence kernel association tests (32), and meta-analysis of results from the 2 genotyping platforms were performed using RAREMETAL (33).

For single-variant exome analysis, we set the significance threshold for single-variant association analysis as *P < *6.93 × 10^−7^ (Bonferroni correction for 72 171 polymorphic variants) for analysis of samples with GH responsiveness. The significance threshold for a gene-based test association as *P < *2.5 × 10^−6^ (Bonferroni correction for 20 000 genes). The analyses were carried out under the same phenotypic models as for GWAS.

### Association analysis of previously reported height-associated variants

For each individual, we calculated a genetic polygene score (34) as implemented in PLINK (28), using 697 height-associated loci identified by the GIANT consortium (18). Specifically, the reported regression coefficient of each SNP on height and the individual genotype at each SNP is used to create a weighted polygene score for each individual. We calculated the variance in GH response explained by this polygene score (adjusted R^2^ estimated from a linear regression model incorporating the score as the predictor and the normalized height SDS as an outcome, adjusting for age at GH initiation and gender).

### Association analysis of GHR-d3 and IGFBP3 variants

We tested for association with variants previously reported to be associated with GH response, using the methods described above. For the *GHR*-d3 variant (10), we used as a proxy the SNP rs6873545 (*r*^2^ between GHR-d3 and rs6873545 = 1 in the HapMap CEU European ancestry sample); we imputed the previously reported IGFBP3 rs2854744 variant.

### Validation and replication of variants with suggestive associations to GH response

We used PLINK to “clump” variants into loci based on linkage disequilibrium (*r*^2^ > 0.5), with a single lead variant in each locus selected based on the best *P* value. We selected for additional genotyping 3 variants that had associations *P* < 5 × 10^-7^ in the primary analysis and 5 additional variants with *P* < 5 × 10^-8^ in any secondary analysis, as well as an additional 29 variants with associations *P* < 5 × 10^-7^ in any secondary analysis. We performed Sanger sequencing in a subset of the GWAS samples to confirm good agreement with the original GWAS data before proceeding to genotyping. In total, we obtained genotype data in the Brazilian replication sample for 26 of these 37 SNPs; 5 of the 28 SNPs were monomorphic in the replication sample and were not analyzed further. Genotyping was performed using the Sequenom MassArray platform as previously described (35) or, for a subset of variants that could not be genotyped successfully, by Sanger sequencing.

## Results

**Figure 2.**
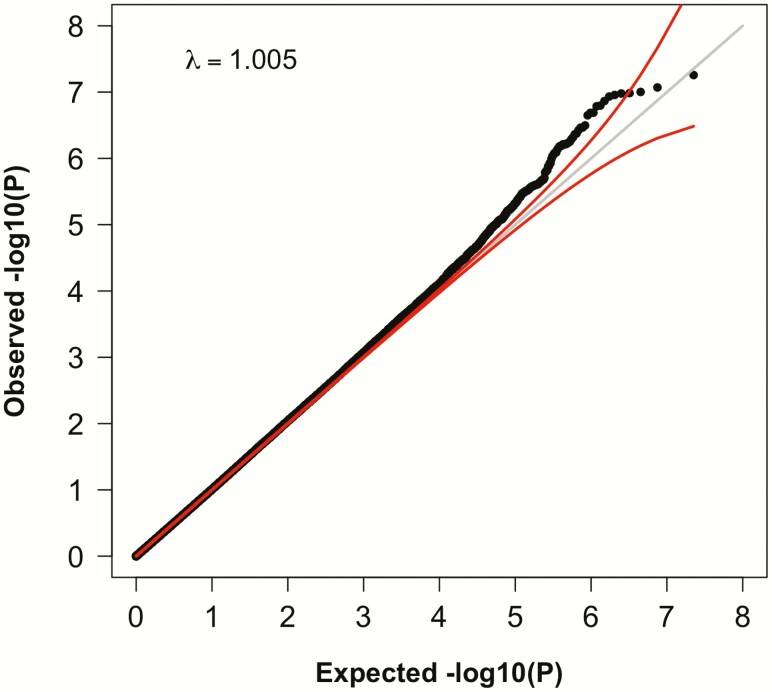
Quantile-quantile (QQ) plot for results from the primary association analysis of common variants with growth hormone response. Each point represents the result from a single variant (genotyped or imputed), ranked by observed *P* value. The x-axis indicates the expected *P* value and the y-axis indicates the observed *P* value, both in negative log scales. The gray line is the expected result under the null, and the red lines indicate 95% confidence intervals around the expectation.

To search for genetic determinants of response to GH, we studied 614 children treated with GH and assembled from several studies (Table 1; see Methods). The participants were categorized as having a diagnosis of GHD (N = 276), SGA (N = 41), or ISS (N = 297). The majority (N = 504) of these samples were of European ancestry. The primary outcome in our study was the increment in height SDS during the first year of therapy. We first performed a GWAS and imputed (26, 27) genotypes for all polymorphic variants with frequency >1% in the 1000 Genomes Phase I Integrated Release Version 3 (March 2012) (25). Our primary analysis included all samples, regardless of diagnosis, and was adjusted for age, sex, and principal components of genetic ancestry (Fig. 1; see Methods). We also performed secondary analyses stratified by ancestry and diagnosis, adjusting for a more extensive set of covariates (maximally adjusted; see Methods). The distributions of test statistics for each analysis were not significantly inflated: genomic inflation correction factors (λ _GC_) ranged between 1.004 and 1.021, indicating minimal systematic bias (Fig. 2).

### GWAS analysis of common variants with GH response

**Figure 3.**
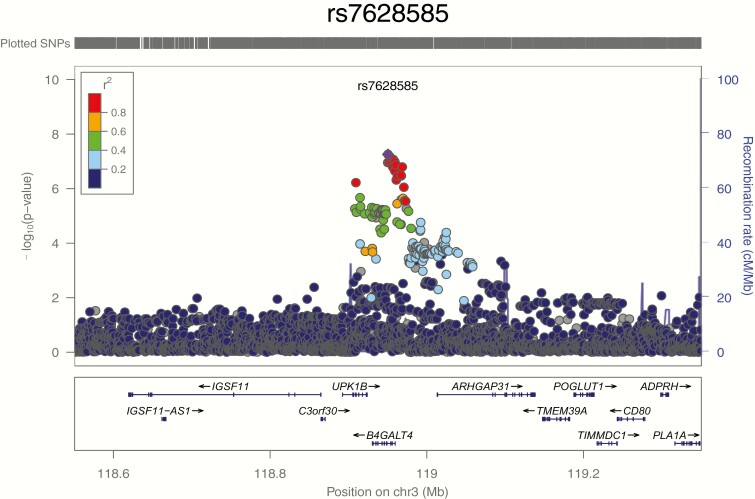
LocusZoom plot for the *B4GALT4* locus. The x-axis indicates position on chromosome 3 surrounding the lead signal of association near *B4GALT4*, rs7628585. Genes depicted in their respective locations. Each dot is a variant in the region tested and is colored by its correlation (r2) with rs7628585 according to the color key. Blue lines indicate recombination rates. The arrow and circle indicate the location and association signal of the second signal at *B4GALT4*, rs35583194; this insertion/deletion variant was not included in the LocusZoom plot because it is not present in the 1000 Genomes reference panel. SNP, single nucleotide polymorphism.

We first tested common variants with minor allele frequency (MAF) above 1% for association with GH response. In our primary analysis (Fig. 1), no variants achieved genome-wide significance (*P* < 5 × 10^-8^; Table 2). The strongest suggestive (*P < *5 × 10^–7^) associations with GH response were observed with a common variant near the *B4GALT4* gene: rs7628585 (intronic, MAF = 0.182; *P = *5.54 × 10^–8^); (Fig. 3) and with a common SNP in the *TBCE* gene, rs1977748 (intronic, MAF = 0.411; *P = *3.75 × 10^–7^).

**Table 2.** Top Associations with GH response from Primary and Secondary GWAS Analyses

Chromosome (position)	SNP ID	MAF	Minor allele	GWAS Effect Size, Best Analysis	GWAS SE, Best Analysis	GWAS *P* value, Best Analysis	Ancestries/ Diagnosis/ Model for Best Analysis	Nearest Gene(s)	Replication Beta	Replication SE	Replication *P* Value	Meta- analysis Beta	Meta- analysis SE	Meta- analysis *P* Value
**Associations from primary analysis with *P* < 5 × 10** ^**–7**^														
3 (118950499)	rs7628585	0.182	A	0.338	0.0621	5.54E-08	Primary analysis	*B4GALT4*	–0.091	0.181	6.17E-01	0.292	0.059	6.47E-07
1 (235531451)	rs1977748	0.411	T	–0.286	0.0563	3.75E-07	Primary analysis	*TBCE*	0.001	0.159	9.96E-01	–0.254	0.053	1.72E-06
**Associations from secondary analyses with *P* < 5 × 10** ^**–8**^														
1 (10194439)	rs55704135	0.03	A	–0.934	0.159	4.24E-09	European/all/ maximum	*UBE4B*	–0.231	0.308	4.54E-01	–0.786	0.141	2.65E-08
3 (98383646)	rs189532746	0.013	A	–1.635	0.282	6.95E-09	All/ISS/ minimum	*CPOX, ST3GAL6*	NP	NP	NP	NP	NP	NP
12 (10300486)	rs78263566	0.025	G	1.685	0.283	8.50E-09	European/ GHD/ maximum	*CLEC7A, OLR1*	0.182	0.721	8.01E-01	1.485	0.2631	1.67E-08
8 (98853066)	rs74523128	0.01	A	2.516	0.430	1.52E-08	European/ GHD/ maximum	*LAPTM4B*	ND	ND	ND	ND	ND	ND
6 (116512418)	rs144751704	0.014	T	–1.299	0.232	2.12E-08	European/all/ minimum	*NT5DC1/ COL10A1*	NP	NP	NP	NP	NP	NP

Chromosome and position are based on the hg37 build of the human genome. Best analysis refers to the ancestry/diagnosis/model with the best *P* value in the GWAS for that variant. Replication and meta-analysis results are shown for that best analysis.

Abbreviations: GWAS, genome-wide association study; MAF, minor allele frequency; ND, not done; NP, not polymorphic; SNP, single nucleotide polymorphism.

In secondary analyses, 5 SNPs had association *P* values below 5 × 10^–8^ (Table 2); all of these had minor allele frequencies below 5%. In light of the sample size of this study, these associations, especially those specific to 1 diagnosis, should be viewed as tentative in the absence of additional replication. A low-frequency intronic SNP (rs55704135, MAF = 3%) in the *UBE4B* gene showed the strongest association to GH response in the analysis of all European ancestry individuals under the maximally adjusted model (*P* = 4.2 × 10^–9^). An intergenic SNP (rs189532746, frequency = 1.3%) between *CPOX* and *ST3GAL6* showed its strongest association in the analysis of individuals with ISS of all ancestries under the minimally adjusted model (*P = *6.95 × 10^–9^). An intergenic SNP (rs78263566, frequency = 2.5%) between *CLEC7A* and *OLR1* and an intronic SNP (rs74523128, frequency = 1.0%) in *LAPTM4B* showed strongest associations in the analyses of individuals with GHD under the maximally adjusted model in European ancestry individuals (*P = *8.5 × 10^–9^ and *P = *1.5 × 10^–8^). Finally, a low-frequency intronic SNP (rs144751704, MAF = 1.4%) located in the *NT5DC1* gene and upstream of *COL10A1* showed the strongest association to GH response in the analysis of all European ancestry individuals under the minimally adjusted model (*P* = 2.1 × 10^–8^). We also identified 29 additional variants with *P* < 5 × 10^–7^ in secondary analyses, a level of significance suggestive of association.

### Attempted replication of potential signals of association with response to recombinant GH

We first focused on the 2 signals that reached suggestive significance in our primary analysis: rs7628585 near *B4GALT4* and rs1977748 near *TBCE*. Neither of these signals were replicated nor reached genome-wide significance (Table 2). We also attempted to replicate the 5 signals that had reached genome-wide significance in a secondary analysis, meta-analyzing the discovery and replication samples using either the same model for which we observed the best evidence for association in the discovery sample or using the primary analysis (minimal covariates, all diagnoses) to maximize power. Two signals for which we were able to obtain replication data retained genome-wide significance after meta-analysis (rs55704135 near *UBE4B*; meta-analysis *P* = 2.65 × 10^–8^ in the European ancestry/all diagnoses/maximum covariate analysis, and rs78263566 near *CLEC7A*; meta-analysis *P* = 1.67 × 10^–8^ in the European ancestry/GHD/maximum covariate analysis, Table 2). However, for both of these signals, the evidence for association decreased after replication, so further validation of these associations would be needed. Two other genome-wide significant variants, (rs189532746, between *CPOX* and *ST3GAL6*, and rs78263566, between *CLEC7A* and *OLR1*) were not polymorphic in the replication analysis, and we were unable to successfully genotype the fifth signal (rs144751704, located in *NT5DC1* and upstream of *COL10A1*) in the replication sample.

We also attempted to replicate 29 signals with suggestive signals in secondary analyses. Of the 19 signals that were successfully genotyped, 12 were polymorphic in the replication sample, and 2 achieved genome-wide significance in meta-analysis with the replication data when analyzed under the same model as was highlighted by the discovery sample. These 2 signals were rs75922185 near *MGAT5*; meta-analysis *P* = 4.93 × 10^–8^ in the European ancestry/all diagnoses/maximum covariate analysis, and rs151058087 near *AEBP2*; meta-analysis *P* = 1.67 × 10^–8^ in the all ancestry/ISS/maximum covariate analysis (Online Supplementary Table 1) (36). The variant near *AEBP2* was the only one to achieve nominal significance in the replication sample. Of potential interest, rs115307564 near *RBMS3*, strongly replicated under the primary analysis model (replication *P* = 7.96 × 10^–4^, meta-analysis *P* = 1.67 × 10^–6^; Online Supplementary Table 1) (36). This variant therefore represents another promising signal of association.

### Association analysis of rarer coding variation with response to recombinant GH using exome array genotypes

To determine the role of low-frequency coding variants, we also analyzed directly genotyped coding SNPs present on the exome array platforms. In single-variant analysis, no SNPs were significant after correcting for multiple testing (see Methods). Because single-variant tests are often underpowered to detect associations with rare variants, we also carried out gene-based association tests using missense, splice, and loss-of-function variants within each gene (see Methods), combining information from the different variants in each gene using the sequence kernel association test (32), and limiting analysis to variants below 1%. No genes were significant after correcting for multiple testing (see Methods). A full set of association results for our primary analysis for all variants with MAF above 1% and all directly genotyped coding variants will be made available.

### GWAS analysis of variants previously associated with GH response or adult height

We also focused specifically on candidate polymorphisms previously associated with response to GH, including the *GHR* d3 variant (10-12), which is represented in our study by the highly correlated SNP rs6873545 (37), as well as the rs2854744 polymorphism in the IGFBP-3 promoter (13, 38). We confirmed that the rs6873545 was a good proxy for the *GHR* d3 variant in our study (see Methods). We observed no evidence at either of these variants for association with response to GH (*P* > 0.05 under all models). Thus, we could not replicate the previous associations.

We also tested a hypothesis that genetic predisposition to tall or short stature could influence response to GH. For each individual, we constructed a genetic polygene score based on the previously established 697 height variants and tested for association between this polygene score and the response to GH. We did not see even nominally significant evidence of association, suggesting that genetic control of final height and response to GH are not strongly connected.

## Discussion

We report what is, to our knowledge, the largest GWAS of response to GH to date. We leveraged the large sample size to interrogate both a primary analysis (all samples, with a minimal set of covariates) as well as several secondary analyses, including testing for association in all samples and in European samples only, with specific diagnoses (ISS and GHD), and adjusting for a larger set of known clinical covariates (gestational age, age at start of GH therapy, GH dosage, height SDS at start of GH therapy, MPH).

We detected 37 suggestive associations (*P* < 5 × 10^–7^) from this GWAS, including 3 suggestive associations from our primary analysis and 5 that had *P* values below 5 × 10^–8^ in at least 1 secondary analysis. The strongest of the associations in the primary analysis was with common variation near the *B4GALT4* and *TBCE* genes. However, despite these strongly suggestive genetic data, we did not observe associations with GH response in our replication sample. Possible explanations for the lack of replication include lack of power in the replication sample, differences in ethnicity (and hence different levels of linkage disequilibrium with an untyped causal variant), or that both of these suggestive associations are statistical fluctuations rather than true associations. Interestingly, the *B4GALTT4* gene is 1 of 7 beta-1,4-galactosyltransferase (beta4GalT) genes, and mutations in *B4GALT7* lead to abnormal skeletal growth (39). However, mutations in *B4GALT4* have not yet been reported to be associated with any specific disease. Of interest, a low-frequency variant that reached genome-wide significance in a secondary analysis is located near *ST3GAL6*, a sialyltransferase that acts in the same pathway as the beta4GalT genes. The frequency of this variant was too low to robustly perform replication.

Of the remaining variants that we were able to assay in the replication sample (see Methods), 4 signals had meta-analysis *P* values that reached or retained genome-wide significance in secondary analysis (near *UBE4B*, *CLEC7A*, *MGAT5*, and *AEBP2*). Remarkably, *MGAT5* also encodes a glycosyltransferase, although not obviously in the same pathway as the beta4GalT genes. Thus, our results provide an intriguing suggestion that variation in glycosylation pathways may regulate the response to GH.

Another low-frequency variant, near *RBMS3*, was the only one to show a strong signal in the replication cohort primary analysis but did not reach genome-wide significance. RBMS3 encodes a long noncoding ribonucleic acid that may modulate signaling by transforming growth factor beta, a well-known modulator of growth; of note, the *TGFBR2* gene is adjacent to *RBMS3.* This variant, as well as the variants described in the previous paragraphs, represents signals that would benefit from further validation to see if they are robustly associated with GH response.

We also tested variants previously associated with response to recombinant GH (rhGH), including the exon 3 deletion variant in *GHR* (10-12) and the rs2854744 and rs924140 variants in *IGFBP3* (13, 38). We found no evidence of association with response to rhGH for any of these variants (all *P* > 0.05). One possibility why we failed to see an association in our large sample is that the findings from the prior studies are false-positives. We note that our study population is a heterogeneous collection of patients with short stature, so differences in patient population could also explain these discrepant findings. However, we also saw no evidence of association when we restricted our analysis to patients with ISS or to those with GHD.

We also tested whether the 697 height-associated loci are associated with response to rhGH. We found no strong association with GH response for any height-associated loci, either individually or jointly. One possible explanation is that the GH response is regulated by different biological mechanisms than for height; another possible explanation is that variation in GH response is associated with variants that are rare and have not been identified from previous GWAS analyses. Comprehensive sequencing of samples with data on GH response could help address these possibilities. For example, if a fraction of patients treated with GH have rare, nearly mendelian causes of short stature, then the variable response to GH could be partly dictated by the variable underlying etiologies in these patients.

Limitations of our study include the collection of samples from various locations in the world with various ethnic backgrounds, and a mixture of diagnoses. We addressed this heterogeneity by performing secondary analyses of more homogeneous, if smaller, subgroups of individuals. The sample size is larger than previous studies but still quite small as compared with many GWAS studies and, therefore, has limited power to detect variants associated with GH response. A sample size of 614 should have 80% power to detect associations that explain, in our heterogeneous population, 6.5% of the variance in GH response. Although effect sizes this large are unusual for polygenic traits, large effect sizes have been seen in other pharmacogenomics scenarios. Thus, any heritable contribution to GH response is likely polygenic. While our work shows that there are likely no common variants that on their own have clinically significant predictive power, larger sample sizes would be needed to identify variants with smaller effects that in combination, as part of a polygenic risk score, could still provide meaningful prediction of the response to GH.

Our study only examined the relationship between genotype and growth response. As noted earlier, other approaches, such as gene expression profiling, can look at other molecular predictors of GH response, and these alternate approaches have been reviewed elsewhere (40). For example, 1 study looked at the methylation status of the IGF-1 promoter and found that approximately 25% of the first-year GH response may be attributable to differences in methylation at the P2 promoter of IGF-1 (41). Additionally, while our study focused on first-year height response, consideration must be given to other definitions of GH responsiveness including biochemical, transcriptomic, and proteomic responses, which may offer more mechanistic insights into GH biology.

In summary, we have completed the largest genetic study to date of response to GH. We were unable to replicate previous associations, nor do we identify any new variants that are clearly and robustly associated with GH response. However, some associations reached genome-wide significance in secondary analyses and merit further investigation, and our data collectively raise the hypothesis that variation in glycosylation may contribute to variation in GH response. Larger sample sizes will be needed to more definitively identify any genetic factors that robustly influence the response to GH.

## Data Availability

The datasets generated during and/or analyzed during the current study are not publicly available but are available from the corresponding author on reasonable request.

## Abbreviations

beta4GalT: beta-1,4-galactosyltransferase
GH: growth hormone
GHD: GH deficiency
GWAS: genome-wide association study
HE: HumanExome
HO: Illumina HumanOmni1-Quad BeadChip
HOEE: HumanOmniExpressExome
IGF: insulin-like growth factor
ISS: idiopathic short stature
MAF: minor allele frequency
MPH: midparental height
rhGH: recombinant GH
SDS: standard deviation score
SGA: small for gestational age
SNP: single nucleotide polymorphism

## Additional Information

***Disclosure Summary:*** A.D. has received consulting fees from OPKO Biologics, Sandoz, Novo Nordisk, Ipsen, and Pfizer; A.A.L.J. received speaker fees from Sandoz and NovoNordisk; K.A.W. received an unrestricted grant from Pfizer; M.B.R. has received speaker fees from Novo Nordisk, Merck Serono, Sandoz and Mediagnost. J.C., M.P.W., and C.C.H. are employees of Pfizer Inc; A.L. was an employee of Pfizer Health AB; Y.M. is now an employee of Fulgent Genetics; J.N.H. received research support from Pfizer Inc for this study and is on the scientific advisory board of Camp4 Therapeutics; S.V., B.W., L.A., and A.C. have nothing to declare.
